# High parasite prevalence driven by the human-animal-environment interface: a One Health study in an urban area in southern of Chile

**DOI:** 10.3389/fvets.2025.1536861

**Published:** 2025-03-21

**Authors:** Daniel Sanhueza Teneo, Omar Cerna, Cédric B. Chesnais, David Cárdenas, Paula Camus

**Affiliations:** ^1^Facultad de Medicina, Instituto de Inmunología y Parasitología, Universidad Austral de Chile, Valdivia, Chile; ^2^TransVIHMI, Montpellier University, INSERM Unité, Institut de Recherche pour le Développement (IRD), Montpellier, France

**Keywords:** parasites, zoonosis, public health, One Health, Chile, pathogens

## Abstract

Parasitic infections remain a global health concern, affecting human populations worldwide. However, comprehensive studies evaluating human, animal, and environmental interactions driven transmission of parasites are limited. We conducted a One Health study in an urban area of Valdivia, Chile. Human participants provided fecal and blood samples for parasitological and serological analysis. Environmental soil samples were collected from public parks, and fecal samples from owned and stray dogs were analyzed. Detection of intestinal parasites employed microscopy and molecular techniques, including next-generation sequencing (NGS), while anti-Toxocara canis antibodies in humans were assessed using ELISA. Socioeconomic surveys explored risk factors associated with parasitism. Parasite prevalence was 28% in humans, 26% in owned dogs, and 44% in environmental dog feces. Anti-*T. canis* IgG antibodies were present in 33% of humans. Soil contamination was identified in up to 30.5% of park samples, harboring zoonotic parasites such as *Toxocara* sp. and *Trichuris vulpis*, the same species identified in environmental dog feces. Zoonotic subtypes of *Giardia duodenalis* and *Blastocystis* sp. were detected in humans. Our findings highlight significant zoonotic and environmental transmission contributing to human parasitic infections in urban settings, underscoring the need for integrated public health interventions. This study demonstrates the importance of adopting an OneHealth approach in the study of parasitology. The complex ecology of parasites requires an integrated perspective to fully understand their transmission pathways and develop effective control strategies. By emphasizing the interconnectedness of human, animal, and environmental health, we aim to contribute to the management and mitigation of this persistent public health issue.

## Introduction

Parasitic diseases remain a major global health challenge, affecting at least one in six people worldwide, with the highest burden in economically vulnerable regions (1). Many of these infections are classified as Neglected Tropical Diseases (NTDs), disproportionately impacting marginalized populations and reinforcing cycles of poverty (2).

Parasite transmission is influenced not only by host-pathogen interactions but also by environmental and urban conditions. Climate change, for instance, alters key factors such as temperature, humidity, and precipitation, potentially facilitating parasite persistence and spread in both endemic and non-endemic regions (3, 4). Among intestinal parasites, protozoa such as *Blastocystis* sp., *Giardia duodenalis* (syn. *Giardia lamblia*), *Cryptosporidium* spp., and *Entamoeba* spp. are widely distributed and primarily transmitted through contaminated food and water (5–7).While more prevalent in developing countries, these parasites are also detected in developed nations, often linked to contaminated water sources, travel, or migration (8). Risk factors for infection include inadequate sanitation, poor water quality, and contact with infected animals, many of which act as reservoirs for zoonotic parasites (5, 7).

Although sanitation improvements have significantly reduced intestinal parasite prevalence, alternative transmission routes must be considered. Studies in Chile and Argentina have shown that prevalence rates range from 37.5 to 50.7% in populations with access to drinking water, rising to 68.1–92.9% in those without (9, 10). In Chile, a long-term study in Talca documented a decline in pathogenic parasites like *G. duodenalis* (from 18.5 to 5.5%) and *Ascaris lumbricoides* (from 10 to 0.1%). However, a dramatic increase was observed in the prevalence of *Blastocystis* sp., from 7.6% (1990–1994) to 73% (2005–2007), probably associated with an improvement in its diagnosis (11). Similar trends have been observed in Valdivia and Puerto Montt, where *Blastocystis* sp. and *Entamoeba coli*—both with zoonotic potential—are highly prevalent (10, 12).

Dogs, particularly strays, are key reservoirs of zoonotic parasites and contribute to environmental contamination. Studies in Chilean cities reveal high levels of soil contamination with parasite-laden dog feces: in Los Ángeles and Temuco, 60% of soil samples harbored eggs of *Toxocara* sp., *Ancylostoma* sp., *Dipylidium caninum*, and *Taenia* sp. (13, 14). In areas near Valdivia, such as Niebla and Corral, contamination rates reach 85.1 and 92.3%, respectively (15). One of the most concerning parasites is *Toxocara canis*, a nematode found in dogs that can cause visceral and ocular larva migrans in humans (16). Diagnosis is typically based on antibody detection, with reported seroprevalence rates reaching as high as 50.6% (17). In 2012, a 25.4% seroprevalence of antibodies against *Toxocara* spp. was reported in Niebla, Chile, alongside 15% soil contamination with *Toxocara* spp. eggs in urban and rural areas (18). In Valdivia, *Toxocara canis* eggs were found in 100% of households with dogs (19).

Since parasite transmission occurs at the intersection of human, animal, and environmental health, a holistic approach is essential (20). This study applies a One Health framework to assess intestinal parasite prevalence in humans and dogs, determine *T. canis* IgG seroprevalence in humans, and evaluate environmental contamination in an urban area of Valdivia, Chile. By integrating these components, we aim to provide a comprehensive understanding of parasite transmission dynamics and associated risk factors, generating evidence to support effective control strategies.

## Materials and methods

### Study area

**Figure 1:**
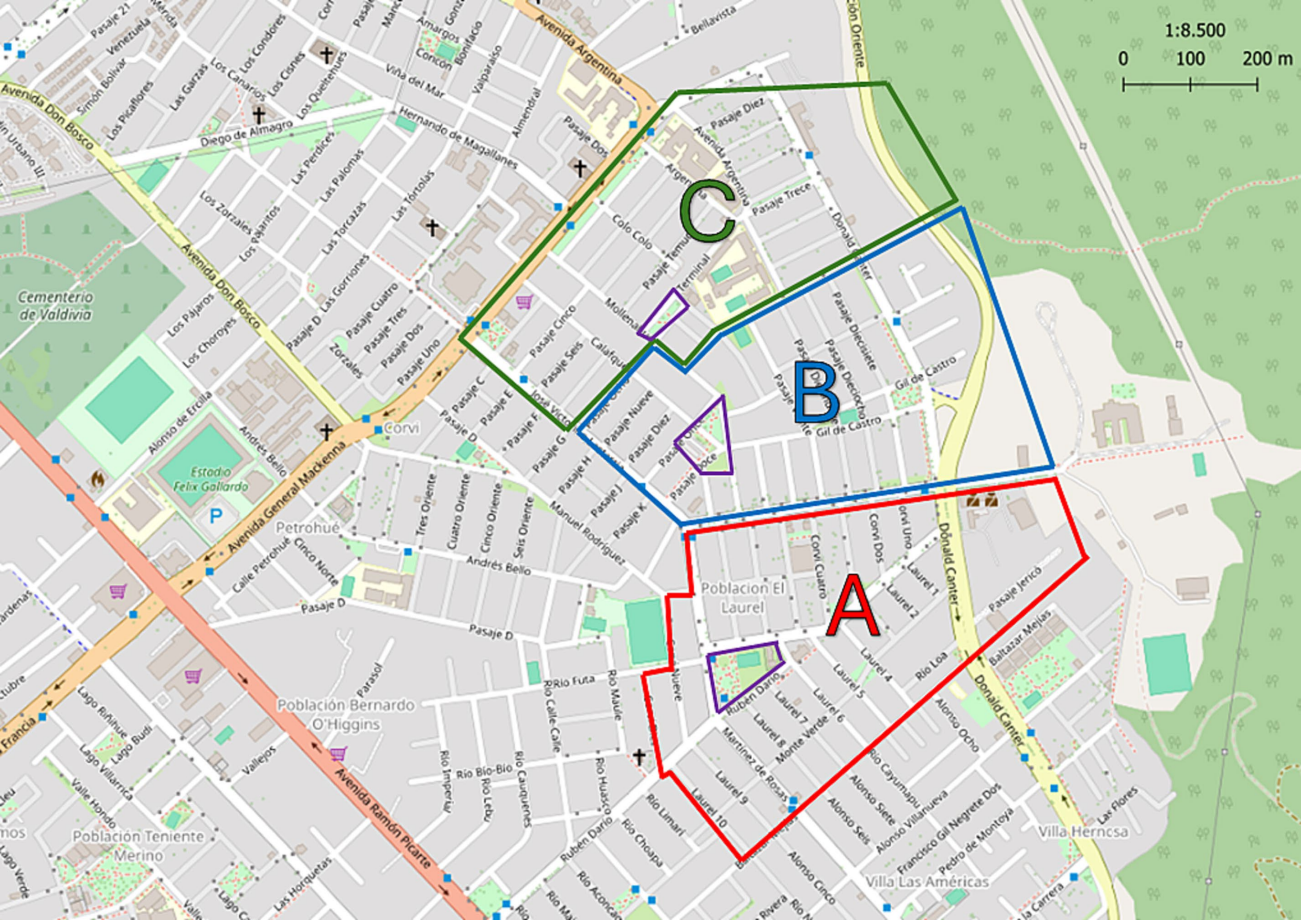
Map of territory covered by Public Health Center “Mulato,” Valdivia, Los Ríos, Chile. The division into three sectors is indicated: A, B, and C with the colors red, blue and green, respectively. The park of each sector is indicated in yellow. Modified from ©OpenStreetMap contributors.

In Chile, public health centers serve a geographic area within each city. The study was conducted at the Community Family Health Center “Mulato” in the city of Valdivia, Chile (39°48′51″S 73°14′45″W), from October 2021 to August 2022. The study obtained the necessary permissions for its realization in humans given by Research Ethics Committee Los Ríos Health Service (Ord. N°354 14.10.2021). Subsequently, to determine the spatial distribution of parasites, the territory was divided into three sectors A, B and C, each with a public park, respectively (Figure 1).

### Human

#### Study population

The estimated population of the study area is approximately 5,000 people. Recruitment was conducted through informational posters at the health center and community meetings. A total of 157 residents participated, providing informed consent for blood and stool sample collection. While all participants provided blood samples, 16 individuals did not return stool samples, resulting in a total of 141 stool samples collected. A survey was carried out on each participant to collect information on their socioeconomic conditions, potentially linked to parasite prevalence (survey details are provided in Supplementary material). Participants, who owned dogs as pets, were offered a diagnostic test to identify intestinal parasites in their dogs.

#### Sample collection

All samples were collected at the health center and then transported to the Institute of Parasitology of the Universidad Austral de Chile. For the diagnosis of parasitosis in fecal samples, each participant received a container with 35 mL of PAF (Phenol, Alcohol, and Formaldehyde) fixative and a 15 mL conical-bottom tube with 8 mL of 70% ethanol, along with oral and written instructions for proper sample collection. Participants deposited fecal samples from three different days (every other day) into PAF containers (21). A single sample from the final collection day was placed in the 70% ethanol tube for molecular analysis. Containers with PAF were stored at 2–5°C, while the ethanol tube were stored at −20°C until processing. To obtain serum, blood samples were collected by health center staff by venipuncture in tubes without anticoagulant and centrifuged at 3,000 rpm for 5 min. The serum was extracted and then frozen at −20°C until the ELISA test was performed.

#### Processing of fecal samples

Fecal samples, from both human and dog, were processed using the Modified Burrows Method (PAFS) (21) and following the recommendations of the National Reference Laboratory of the Instituto de Salud Pública de Chile.

#### Sample processing by ELISA

A commercial ELISA kit (NovaLisa from Novatec) was used to detect IgG anti-*Toxocara canis* antibodies. The test was performed according to the manufacturer’s instructions. Indeterminate results were sent for confirmation to the national reference laboratory at the Institute of Public Health, Santiago, Chile.

#### Analysis of subtypes of *Giardia duodenalis* and *Blastocystis* sp. by using next generation sequencing

Ten human stools samples, previously diagnosed with *Blastocystis* sp. or with *G. duodenalis* by microscopy, were further analyzed using next-generation sequencing (NGS) at the Austral-OMICS laboratory (specialized unit for supporting scientific research). Samples were selected to represent each of the sampling sectors. For *G. duodenalis* the *β-Giardina* gene was targeted, while for *Blastocystis* sp. the 18rRNA gene was analyzed. A detailed description of the technique is provided in Supplementary material.

### Environment

#### Sampling and processing of soil samples

Soil samples were collected from parks A, B, and C during two periods: October 2021 (spring) and May 2022 (autumn). In each park, areas of interest were defined as two meters perimeter the children’s playgrounds, as these locations have the highest presence of both humans and dogs. Within these sampling areas, three parallel imaginary lines were drawn, spanning from one end to the other. Sampling points were established at 1 m intervals and 5 g soil sample was collected from a depth of 3–5 cm. The collected soil samples were dried for 48 h at room temperature. The whole sample was then sieved with a grid into a metal container and processed using the zinc sulfate method (22, 23). For the physicochemical analysis, 300 g of soil from each sector was collected in a re-sealable plastic bag. In the case of parks A and B parks, one sample was taken for each study (parasitological and physicochemical), while in park C there were two soil types, therefore, one sample was taken from each soil type (C1 and C2).

#### Soil physicochemical analysis

The physicochemical analysis of the soil was carried out by the Forest Soil and Nutrition Laboratory of the Universidad Austral de Chile. The parameters measured were pH, % Total Carbon (TC), % Soil Organic Matter (SOM) and % Humidity.

### Dogs

#### Sampling and processing of dog fecal samples collected in the environment of the sector

A walking tour of each of the streets and passageways in the sector was conducted twice, in October 2021 (spring) and May 2022 (autumn). All samples of dog feces that were not dry for collection were obtained in each sector. For each sample, 5 g of stool were placed in containers with 15 mL of PAF fixative. Samples were then transported to the laboratory and processed using the Modified Burrows Method (PAFS) (21).

#### Sampling and processing dog stool samples collected from owned dogs

Participants who agreed to include their dogs in the research received a container with 15 mL of PAF fixative and were asked to collect 5 grams of feces. Finally, the collected stool samples were processed by the Modified Burrows Method (21).

#### Statistical analysis

A descriptive analysis of the data was performed using Chi-Square and a statistical significance level of 0.05 was established. To determine the magnitude of the observed relationship, the Odds Ratio (OR) was used, and a 95% confidence interval (CI) was established. Graphpad Prism 9 and IBM SPSS Statistics (Statistical Package for the Social Sciences) were used in the analysis of the data obtained.

## Results

### Human

#### Description of the study population

One hundred forty-one people participated in the study, of whom 67.4% were women. The participants age ranged from 5 to 89 years, with an average age of 64 years, with most of them (76.6%) between 50 and 79 years old. In terms of educational level, 35.5% completed basic education (8 years), 47.5% completed secondary education (12 years) and 10.6% completed high education (more than 12 years). In addition, 65.3% of the participants are retired or housewives. In terms of income, 95.8% of the participants declare a monthly income less than or equal to US$ 660. Ninety-two point 9 % of the population declares to have at least one chronic non-transmissible disease. Also, 89.4% of the participants report having consumed only drinking water in the last year and 97.9% having access to sewerage.

#### Prevalence of intestinal parasites

**Figure 2:**
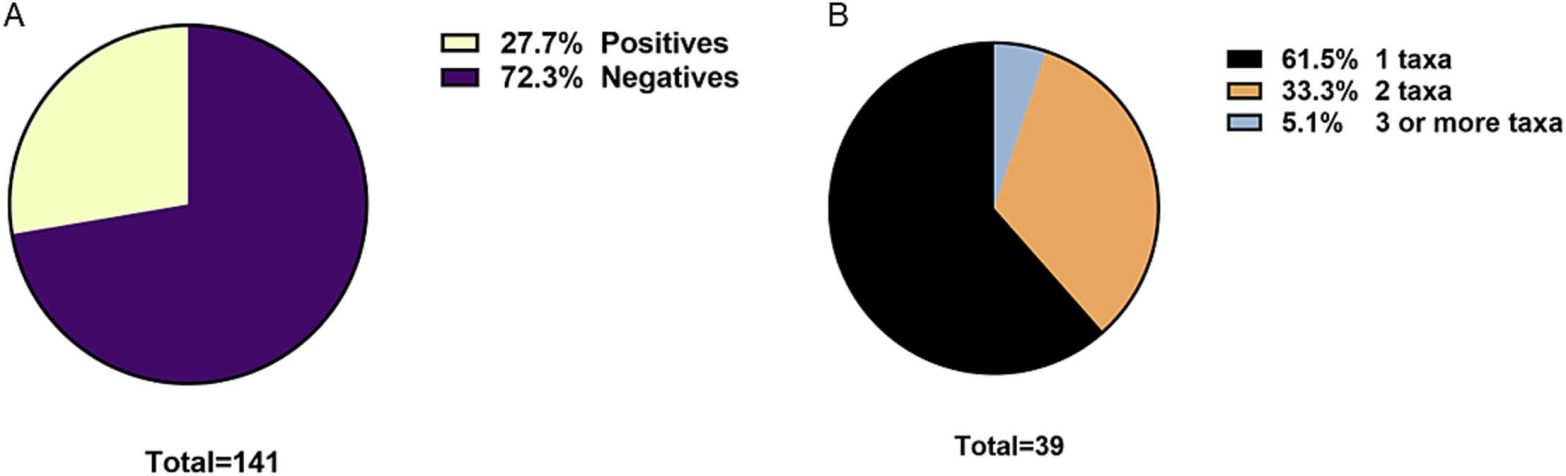
**(A)** Overall prevalence of intestinal parasites in humans. **(B)** Frequency of mono or polyparasitism in humans.

**Figure 3:**
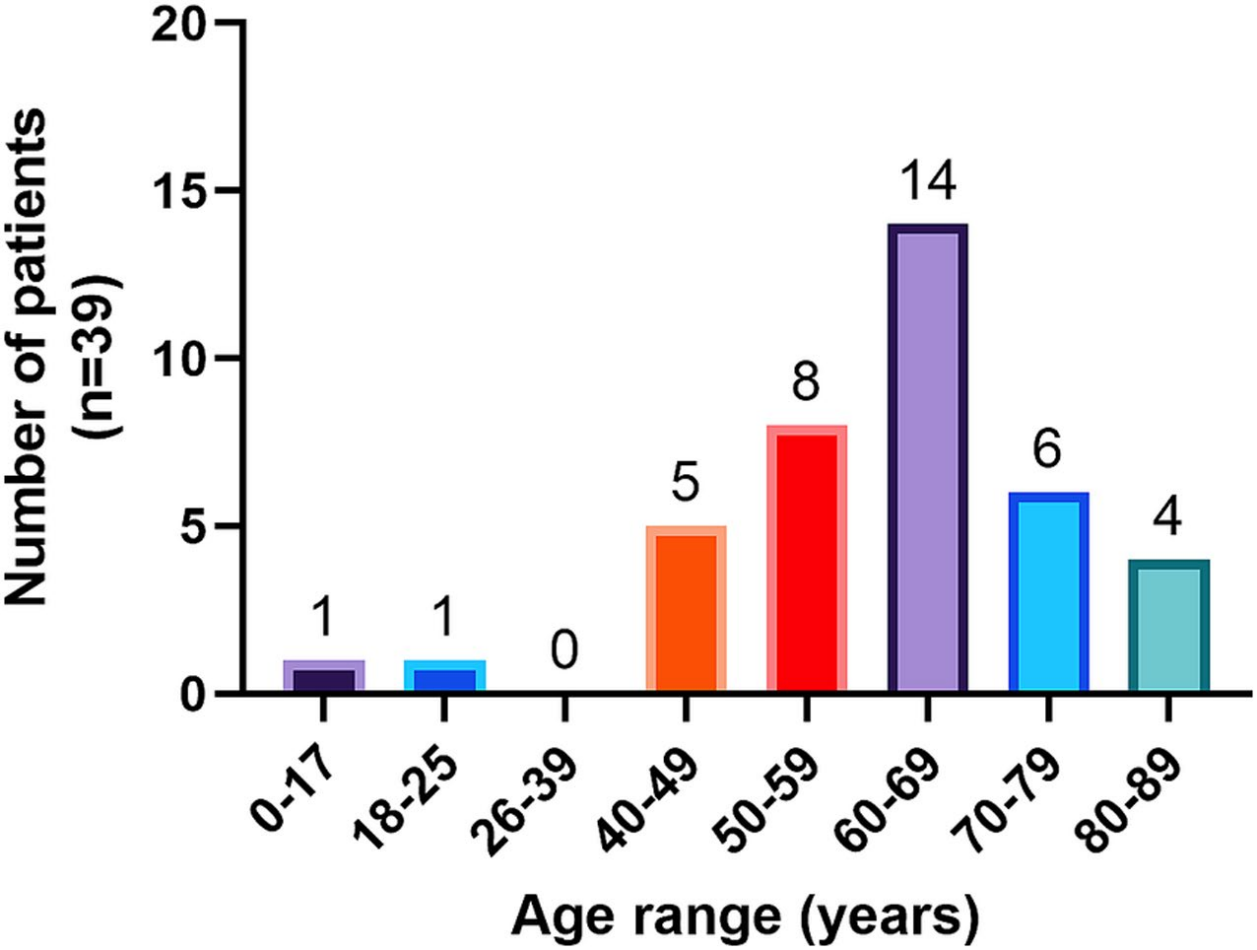
Human patients diagnosed with intestinal parasites by age group.

The overall prevalence of parasites was 27.7% (CI: 20.9–35.6) (Figure 2A). Sixty-one point 5 % of positive patients had 1 parasitic agent, 33.3% had two parasitic agents, and 5.1% had 3 or more (Figure 2B). Among the positive patients, 61.5% correspond to women. The age distribution of patients with parasites shows that the majority correspond to patients over 60 years of age (Figure 3).

**Figure 4:**
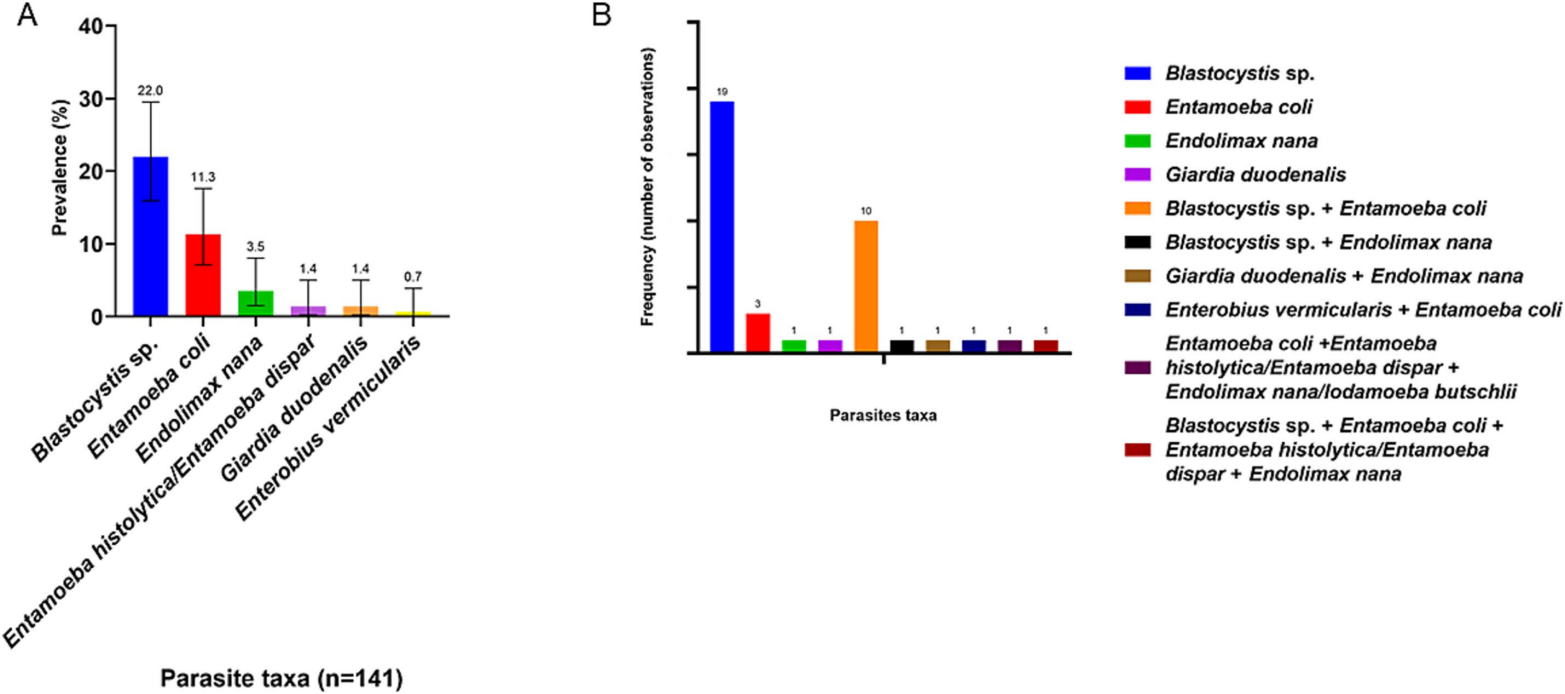
**(A)** Prevalence of parasite taxa in human population. The number indicated above the bars refers to the prevalence of each parasite. **(B)** Frequency of observation of each parasite taxa, whether singly or in association with other parasites.

In terms of prevalence by species or taxa, the most prevalent parasites were *Blastocystis* sp. with 22% (CI: 15.9–29.5), followed by *Ent. coli* with 11.3% (CI: 7.1–17.6) and *Endolimax nana* with 3.5% (CI: 1.5–8.0) (Figure 4A). As previously mentioned, in our study almost 40% of polyparasitism was observed (Figure 2B). Thus, Figure 4B described in detail the frequency of observation of each parasite related to other parasites taxa. *Blastocystis* sp. is the most frequently described singly (19 times) but also accompanied by *Ent. coli* (10 times).

Comparing each of the components of the socioeconomic survey with the presence of parasites, it was found that living with pets is a statistically significant factor (χ^2^ = 4.9, *p* = 0.03). Thus, having pets increases the risk of harboring parasites almost threefold (OR: 2.9, [1.1–7.5]). No statistically significant differences were observed in any of the other socioeconomic factors studied (*p* > 0.05).

#### Prevalence of IgG anti-*Toxocara canis* antibodies

A total of 157 human serum samples were analyzed. The average age of the participants was 61 years, with a maximum of 89 years and a minimum of 4 years. Among them, 110 were women and 47 were men.

There was a global prevalence of 33% of anti-*Toxocara canis* IgG. Thus, 52 participants were positive, 101 negative and four undetermined. The undetermined samples were sent to the Institute of Public Health of Chile, and due to the detection limitations of the ELISA technique used, the result remained indeterminate.

**Figure 5:**
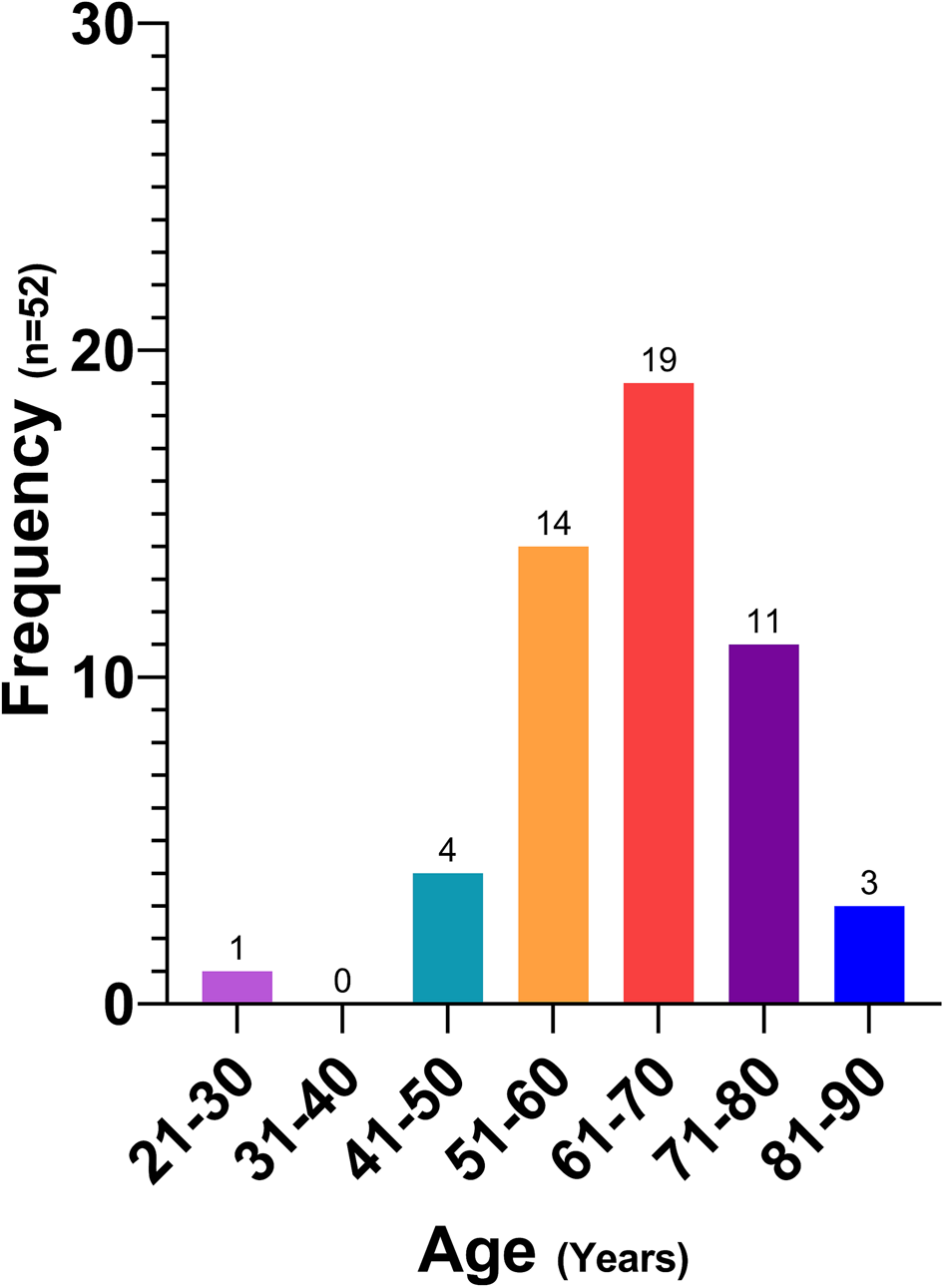
Human patients with a positive ELISA test by age group.

Figure 5 shows the age distribution of the participants who were reactive to the ELISA, with the range between 51 and 80 years showing the highest prevalence with 85% of the cases.

A statistically significant difference was found between the prevalence of anti-*Toxocara canis* IgG and the educational level of the participants (*p* = 0.03), with a lower risk of being serologically reactive at a higher educational level (OR: 0.5; CI: 0.2–0.9).

No statistically significant differences were observed based on gender, income, chronic disease status, outdoor activities, symptoms, living with minors, pet ownership, or having a garden (*p* > 0.05). Similarly, there were no significant associations between anti-*Toxocara canis* IgG prevalence and prior parasite history, treatment, or living with treated individuals (*p* = 0.83), nor with dog ownership (*p* = 0.99) or enteroparasite presence (*p* = 0.65).

#### Subtypes of *Giardia duodenalis* and *Blastocystis* sp. by using next generation sequencing

The samples analyzed by NGS correspond to samples positives by microscopy to *G. duodenalis* or *Blastocystis* sp. In 8 of the 10 samples, there was amplification, and the resulting sequences were compared with database built based on the sequences available in NCBI. For both parasites only one subtype was identified in each sample.

**Figure 6:**
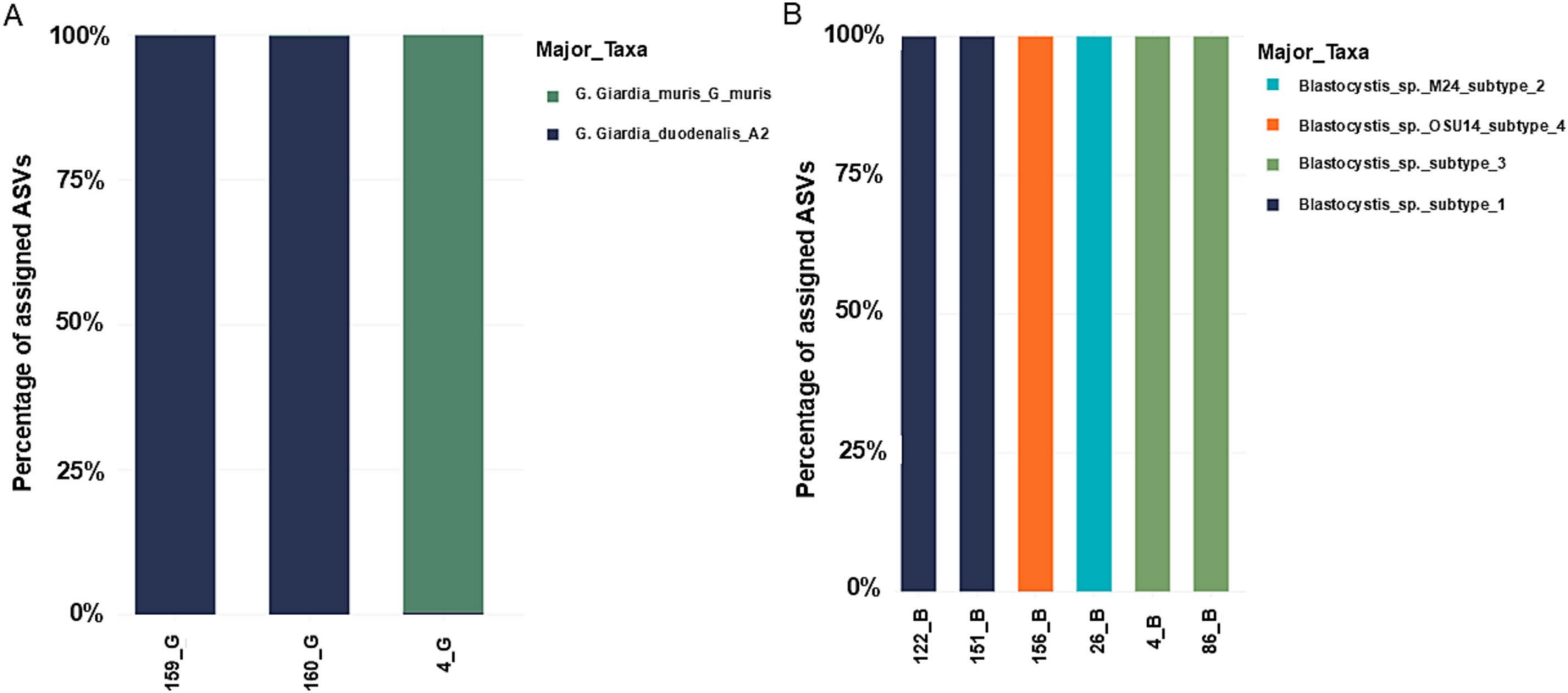
Taxonomic mapping using the database built based on the sequences available in NCBI. **(A)** *Giardia duodenalis*. **(B)** *Blastocystis* sp. Note that in the case of *Giardia duodenalis*, due to the small number of readings of subtypes D and E, they are not observable in the graph.

Three samples were sequenced for *G. duodenalis*, in two of them the A2 assembly was identified (~140,000 reads each). In the third, *G. muris* was identified, a parasite usually described in rodents, but with a very low number of reads (9,364 reads). However, due to the technique used in both samples identified as *G. duodenalis* assembly A2, a marginal number of reads corresponding to *G. duodenalis* assemblies D and E was also observed (~130 reads each), both corresponding to less than 0.1% of the total reads (Figure 6A).

For *Blastocystis* sp. six samples were sequenced with a total of 800,087 reads. Subtype identification was as follows: two samples with subtype 1; two samples for subtype 1, one sample with subtype 2, two samples with subtypes 3 and one sample with subtype 4 (Figure 6B).

### Environment

#### Parasites in soil samples

**Figure 7:**
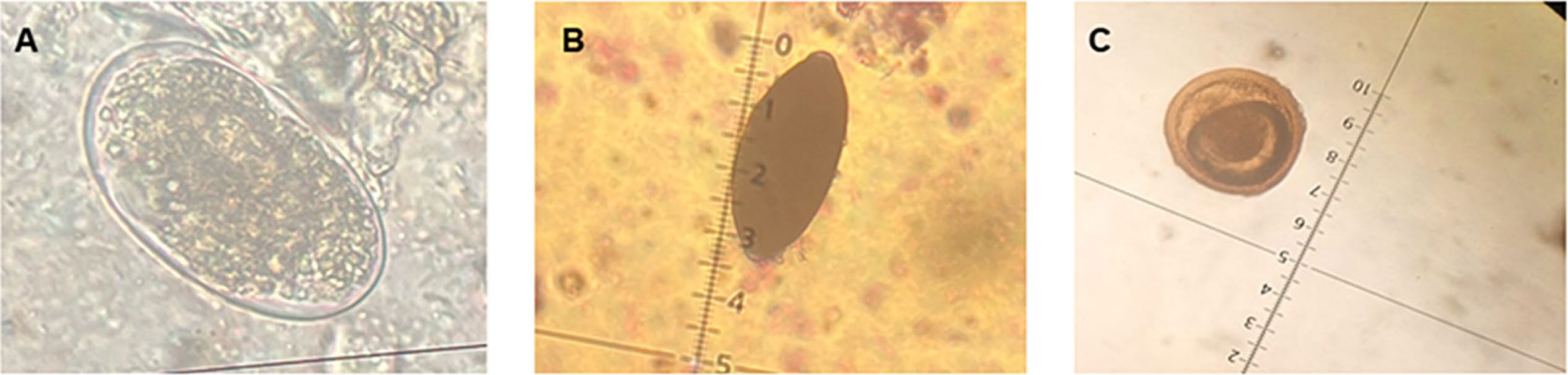
Parasites observed in soil samples. **(A)** Egg of *Uncinaria stenocephala*. **(B)** Egg of *Trichuris vulpis*. **(C)** Egg of *Toxocara* sp. (40x).

A total of 8 soil samples were collected, 4 in spring and 4 in autumn. Of these, 7 contained at least 1 parasitic element. They showed a diversity of parasitic elements composed exclusively of nematode eggs. In spring, eggs of *Trichuris vulpis*, *Toxocara* sp. and *Toxascaris leonina* were found. While, in autumn, eggs of *T. vulpis*, *Toxocara* sp. and *Uncinaria stenocephala* were observed (Figure 7).

The results of the physicochemical analysis of soil samples indicate that parks A and B maintained similar characteristics in total carbon (TC) (%), soil organic matter (SOM) (%) and humidity (%) across both sampling periods. However, the pH in park A increased from 5.2 in spring to 6.3 in autumn. In contrast, significant changes were observed in park C, especially in C1, where TC (%) increased from 4.3% in spring to 8.2% in autumn, and SOM (%) increased from 7 to 14% during the same period.

### Animals

#### Frequency of parasites in dog feces collected in the environment

A total of 180 samples were collected, of which 44.4% (CI: 37.4–51.7) showed the presence of parasites. The seasonal variations show that in spring there were 48.4% and in autumn 40% positive samples. The percentage of positive samples analyzed by sector and per season varied from 16.7% (sector B in autumn) to 79.2% (sector A autumn). In spring no statistically significant difference was observed by the chi-square test (χ^2^) between the frequency of observation of parasitic elements in dog feces and the sector in which the samples were collected (*p* > 0.05). In autumn a statistically significant difference was observed using chi-square (χ^2^) between the frequency of observation of parasitic elements in dog feces and the sector in which the samples were collected. (A vs. B *p* < 0.05, χ^2^ = 18.8 and A vs. C p < 0.05, χ^2^ = 14.2). Additionally, it was observed using the Odds Ratio (OR) that in sector A there was 19 times more probability of finding a positive dog stool sample compared to sector B (OR: 19; CI: 4.4–81.6) and 8.9 times more than in sector C (OR: 8.9; CI: 2.7–30.2). Finally, by performing a chi-square test (χ^2^), it was observed that only in sector A there is a statistically significant difference between the season of sampling (spring or autumn) and the percentage of positive samples (*p* < 0.05, χ^2^ = 4.0). This shows that the spatial distribution of parasites is not homogeneous within the study area.

#### Parasites diversity in dog feces collected from the environment

**Figure 8:**
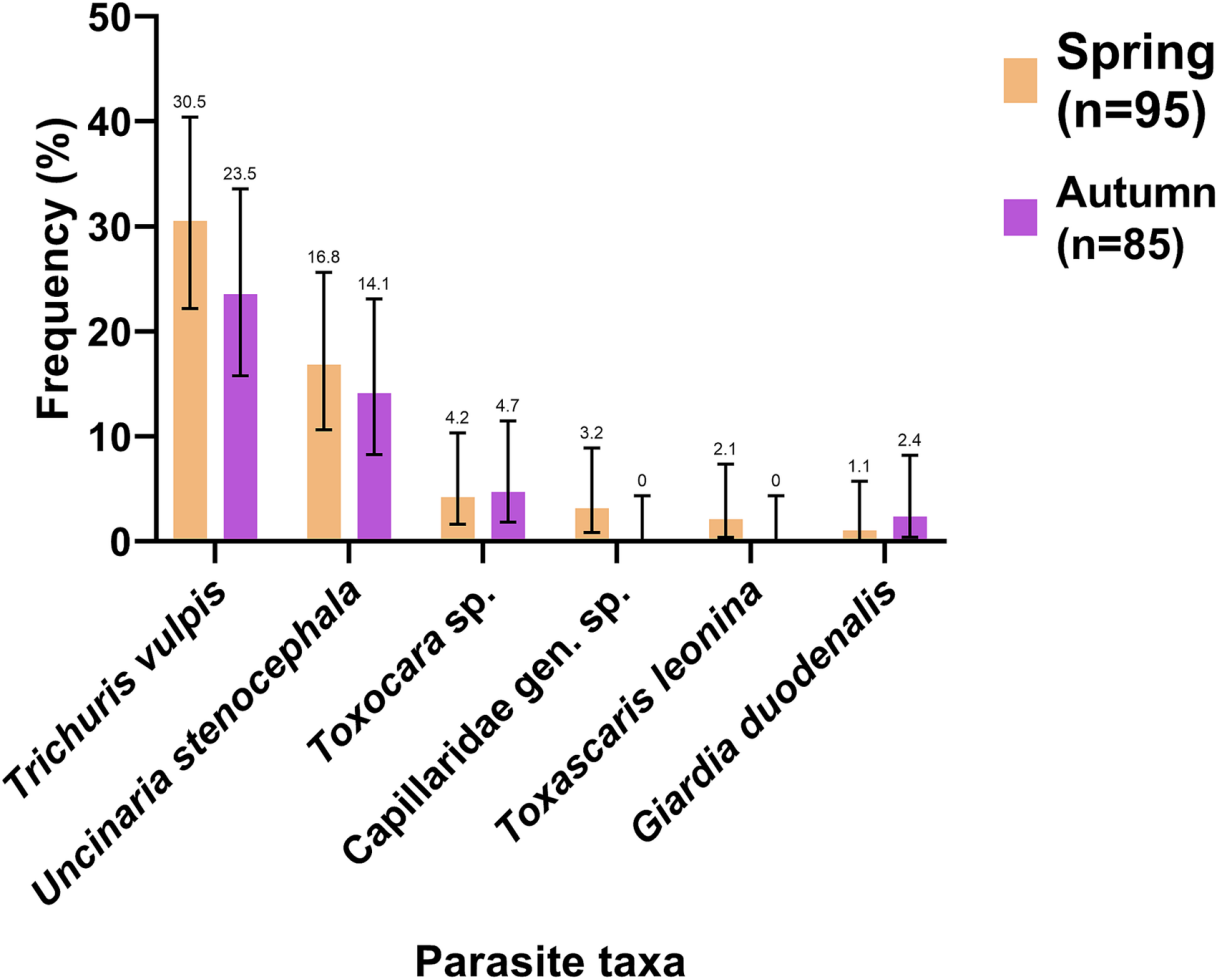
Frequency of observation (%) of parasites in dog feces collected in the environment in spring (orange) and autumn (purple). The number indicated above the bars refers to the frequency of each parasite.

The parasite most present in these samples was *T. vulpis* with 27.2% (CI: 21.2–34.1) of positive samples, followed by *U. stenocephala* with 15.6% (CI: 11.0–21.6). It is important to point out, due to their zoonotic importance and the clinical presentation in humans, the presence of *Toxocara* sp. in 4.4% (CI: 2.3–8.5) and *G. duodenalis* in 1.7% (CI: 0.5–4.8) of the collected samples. Most of the parasitic elements were observed in both spring and autumn, except for *T. leonina* and Capillariidae gen. sp. which were only observed in spring (Figure 8).

According to the sector, *T. vulpis* and *U. stenocephala* were the most frequent species in all sectors (A, B and C). In Sector A, *T. vulpis* was found in 30.6% (CI: 19.5–44.5) and *U. stenocephala* in 18.4% (CI: 10.0–31.4) of the samples. In Sector B, *T. vulpis* was found in 32% (CI: 17.2–51.6) and *U. stenocephala* in 12% (CI: 4.2–30.0) of the samples. In Sector C, *T. vulpis* was found in 28.6% (CI: 13.8–50.0) and *U. stenocephala* in 19% (CI: 7.7–40.0) of the samples. All details about the frequency of parasites found for each sector and period are shown in Supplementary material. When performing the chi-square test (χ^2^), no statistically significant difference was observed between the total positive samples in spring versus the total positive samples in autumn (χ^2^ = 1.3; *p* > 0.05) nor between the sample collection season (spring or autumn) and the frequency of any parasitic species (*p* > 0.05).

#### Parasites in owned dogs

**Figure 9:**
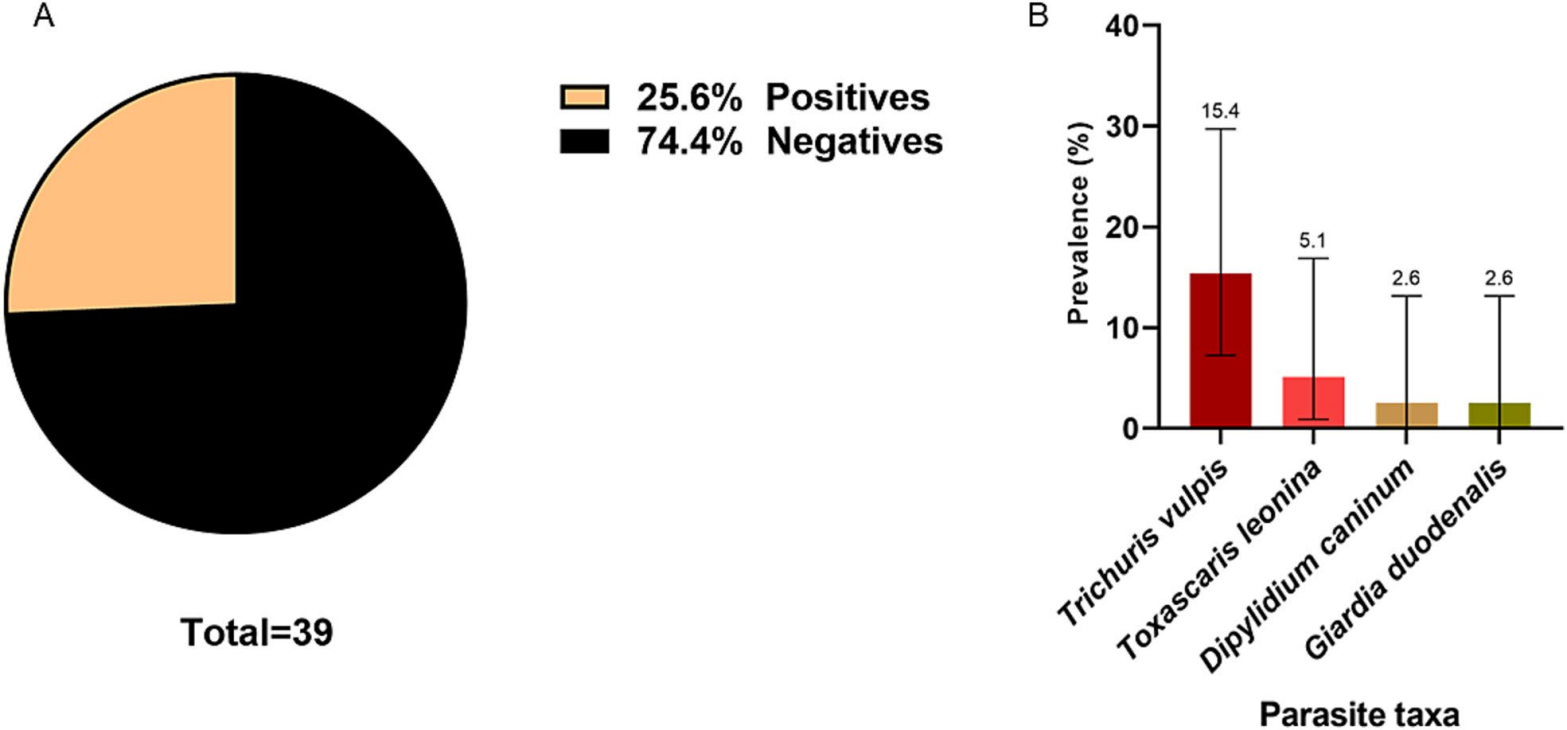
**(A)** Global prevalence of intestinal parasites in samples collected from owned dogs. **(B)** Prevalence of parasite species in samples collected from owned dogs. The number indicated above the bars refers to the prevalence of each parasite.

A total of 38 samples were collected from dogs with owners. A prevalence of 25.6% (CI: 14.6–41.1) was described. Four zoonotic species were observed *D. caninum* (2.6%; CI: 0.1–13.2), *G. duodenalis* (2.6; CI: 0.1–13.2), *T. vulpis* (15.4%; CI: 7.2–29.7) and *T. leonina* (5.1%; CI: 0.9–16.9) (Figure 9).

No statistically significant difference was found between the presence of parasites and whether pets go outside (χ^2^ = 3.3, *p* = 0.07), nor between the presence of parasites and deworming within the last 6 months (χ^2^ = 0.3, *p* = 0.57).

## Discussion

### Human

#### Intestinal parasites in humans

In Chile, recent studies on parasites in the general population are scarce, with the last study in the Los Rios region conducted over 20 years ago. The present study provides an update of the epidemiological data for intestinal parasitosis in humans. Thus, the global prevalence of intestinal parasitism observed in this study was 27.7%, a lower prevalence compared to previous studies in Chile, such as the 1997 study in Santiago, which reported a prevalence of 37.7% (24).

*Blastocystis* sp. was the most prevalent agent identified (22%), followed by *Ent. coli* (11.4%). In contrast to our results, a previous study carried out in Valdivia in 1987 reported higher prevalences, with 61.8% of *Blastocystis* sp. and 30% of *Ent. coli* (12). While *Ent. coli* and other non-pathogenic organisms identified directly cause disease, their presence indicates exposure to fecal contamination, often through contaminated food or water with feces. This is relevant despite high levels of potable water and sanitation services in Chile. Compared to other Latin American countries, Chile demonstrate a lower prevalence of intestinal parasites and polyparasitism. A possible explanation for this could be related to the quality of water sanitation (25). To support this claim, a study analyzed the Environmental Performance Index (EPI) in the drinking water category of 180 different countries. The results concluded that Chile is the leader in Latin America in sanitation and drinking water quality (26). However, even if our study demonstrated a lower prevalence of intestinal parasitosis and polyparasitism in Chile compared to past data and neighboring countries, likely reflecting improvements in sanitation and water quality, the persistence of intestinal protozoa like *Blastocystis* sp. and *Ent. coli* underscores ongoing exposure to fecal contamination, suggesting the need for continued public health efforts.

The causes of enteroparasitosis are multifactorial, influenced by factors such as basic sanitation, drinking water sources, overcrowding, environmental temperature, among others. These factors can significantly impact the probability of parasitosis and explain the variability in prevalence report across different studies, which are highly dependent on the characteristics of the population studied (27, 28). At the local level, the difference in prevalence described in a study performed in 1987 and the present study may be attributed to contrasting living conditions. The 1987 study focused on a rural population, where many lacked accesses to potable water, sanitary excreta disposal, and regulated garbage collection, conditions that are not present in the urban population of this study. Here, 98% of participants reported having access to sewerage system, 91% use only drinking water over the past year and 100% had garbage collection services (12).These improved conditions suggest that the parasitized patients in our study would likely acquire parasitosis through alternative routes, such as the consumption of poorly washed vegetables or coming into contact with soil contaminated with infective stages (28). Furthermore, the statistical analysis associating the presence of parasites with socioeconomic factors showed that living with pets is a statistically significant factor (χ^2^ = 4.9, *p* = 0.03). Participants living with pets were found to be almost three times more likely to have parasites (OR: 2.9, [1.1–7.5]). This highlights zoonotic transmission as the most critical route of infection for intestinal parasites in this urban setting.

#### Prevalence of antibodies IgG anti-*Toxocara canis*

In the present study the prevalence of *anti-Toxocara canis* IgG obtained was 33%. In Chile, a prevalence of 8.8% was previously reported in 1989, 10% in 1998, and 24.4% in 2012 (18, 29, 30). A statistically significant difference was found between the prevalence of *anti-Toxocara canis* IgG and the level of education of the participants (*p* = 0.03) and a higher risk of having anti-*Toxocara canis* antibodies was found with a lower educational level (OR: 0.5; CI: 0.2–0.9). Although education has been associated as a predisposing factor in enteroparasitosis, in toxocariosis its relationship is not clearly established, thus an analysis performed in Pakistan on different human populations, found a statistical association in one group of human and no relationship with others depending mainly with the regular contact of each group with pet animals or livestock, a key factor in the transmission (31). In addition, most serological studies against *T. canis* have focused on children, as they are considered to be the largest population at risk, so education has not been a fully investigated as a factor.

All the other 17 factors studied in our socioeconomic survey showed no statistically significant difference associated to the prevalence of antibodies anti-*Toxocara canis*. Between them, no significant difference was described related to the presence of a garden or patio in the home or with pet ownership (*p* > 0.05). This is consistent with a study conducted in Niebla, Chile, in 2016, which found no statistical association with these variables (18, 32). Studies in Argentina and Venezuela have found statistical significance with dog ownership (33, 34). Since the dog is the definitive host of *T. canis*, this lack of association may indicate that the origin of infection comes from other sources such as the consumption of water or food contaminated with *T. canis* eggs, onychophagia, geophagy, etc. (35). In our study, we observed *T. canis* eggs throughout the sampling period, in urban parks and also in dog feces collected from the environment. These results, associated with the high prevalence of anti-*Toxocara canis* antibodies in humans, allow us to hypothesize that humans are in contact with the parasite from the environment in their own neighborhood and the high presence of the parasite in urban areas (parks and streets) is a source of human infection and should therefore be considered as an important public health issue for local authorities.

#### Subtypes of *Giardia duodenalis* and *Blastocystis* sp. by using next generation sequencing

*Giardia duodenalis* assembly A2 was found in 2 sequenced samples. The A group has zoonotic potential, it is frequently found in companion animals (dogs, cats and horses) and livestock (cattle, sheep, goats, alpacas, pigs, etc.). This group can be divided into subsets, which are A1, A2, and A3. The A2 subset is usually found in human hosts, but has also been found in cats, dogs, horses, and other animals (36–38). Most human infections around the world are caused by the A2 and B genotypes (39). Several studies have reported an association between assembly A and intermittent diarrhea, while other studies have reported a correlation between subset B and severe or persistent diarrhea (40). In this case, the A2 subset found in the samples of our study suggests the possibility of zoonotic transmission involving domestic animals such as cats or dogs.

Regarding the diversity of subtypes in each patient, a single majority subtype was successfully identified in each case. Specifically, one patient had *G. duodenalis* subtype A2 with 137,999 reads, plus a minimal proportion of reads for other subtypes: 165 reads for subtype D and 101 reads for subtype E of *G. duodenalis*. In another patient with *G. duodenalis* subtype A2 (146,476 reads), 87 reads for *Giardia muris* were detected. These readings of other subtypes are attributed to the high sensitivity of the technique used, which can detect even single nucleotide variations, which may result in residual readings representing less than 0.1% of the total readings. These residual readings are not considered reliable for subtype identification, but artifacts generated by the technique. This situation may be due to sequencing errors, chimera formation during PCR, and/or the technology’s high sensitivity. Several strategies could be implemented to mitigate these artifacts in future analyses, such as increasing sequencing depth, applying stricter thresholds to filter out spurious reads, or improving quality controls during sample processing.

Among the samples analyzed by NGS containing *Blastocystis* sp., subtype (ST) 1 was found in 2 samples, 1 sample with ST 2, 2 samples with ST 3 and one sample with ST 4, while in 2 samples (20%) there was no amplification due to the low quality of the DNA extracted. The most common subtypes worldwide are ST 1, 2 and 3, while ST 4 is regularly detected in symptomatic patients in Spain and other European countries, but is rare in South American countries, although it has been previously reported in countries such as Colombia and Brazil (41, 42). Although some studies indicate that *Blastocystis* sp. may be associated with diarrhea, abdominal pain, irritable bowel syndrome, constipation and abdominal distension, it has not been determined whether these symptoms belong to a specific ST, it has also been described that subtypes 1 and 3 of *Blastocystis* sp. are frequently found in patients with irritable bowel syndrome (IBS). whose prevalence is not well documented in Chile. The 4 subtypes present in this study have also been detected in animals such as dogs, pigs, cows, rodents and others (41). In our study, most of the identified subtypes of *Blastocystis* sp. and *G. duodenalis* found in humans are potentially zoonotic parasites, which is consistent with the previously discussed finding that ownership domestic animal increases the risk of having intestinal parasites threefold.

### Environment

#### Parasites in soil samples

Eggs of *T. canis, U. stenocephala, T. vulpis*, and *T. leonina* were found in the surveyed parks; all of which are associated with zoonotic transmission. The contamination of public spaces with parasitic elements is most likely due to the large number of stray dogs observed in the sector. Dogs can excrete up to 600 grams of feces per day on average and cover distances of up to 15 km per day (43), so it is very likely that they have had a significant impact on soil contamination. Similar findings were reported in a study carried out in Temuco (Chile), where eggs of *Toxocara* sp. and *Trichuris* sp. were found. However, in contrast to our study, the presence of *Taenia* sp. eggs was detected (14). This last difference could be related to differing levels of interaction between peri-urban or rural and urban environments (44). In urban settings, the dissemination of cestodes parasitic elements occurs mainly through the feces of carnivorous animals, such as stray dogs (45).

Interestingly, a greater diversity of zoonotic parasitic elements, particularly nematodes, was observed in spring compared to autumn. This is consistent with findings in New Zealand, where soil nematode diversity is higher during the spring season (46). The increased diversity of parasitic nematodes can be attributed to the improved weather conditions, which increases circulation of dogs in public areas, raising the possibility of soil contamination and the potential for parasite transmission (47, 48).

The physicochemical analysis of the soil showed that the pH range was from 5.3 in spring to 6.4 in autumn. The infective stages of geohelminths tolerate soil pH ranges between 4.6 and 9.4 (49). Other studies conducted in Ghana and Egypt have described a significant relationship between soil pH and the number of parasitic stages concluding that the highest number of parasitic elements are found in soils with pH between 5 and 8 (50, 51). Also, in the present study have been described a concentration of total carbon up to 14%, and it has been demonstrated that soils containing a higher amount of total carbon and soil organic matter are associated with a higher number of geohelminth eggs because it affects the porosity of the soil (52), therefore, species such as those belonging to the Ancylostomatidae family are favored in their development due to a soil with greater porosity that allows the infective larva to be kept close to the surface, providing greater oxygenation and facilitating contact with a susceptible host (53). Overall, the results suggest that the parks in our city possess physicochemical properties conducive to the survival and development of various zoonotic parasite species, highlighting the need for targeted public health measures.

### Dogs

#### Intestinal parasites in owned dogs

In the study of feces of dogs with owners, a total of 39 samples were analyzed, revealing an overall prevalence of parasitosis of 25.6%. Among these, *T. vulpis* was observed in 15.4% of the samples, while *G*. *duodenalis* was found in 2.6%. Similar studies conducted in Chile, specifically in the cities of Talca and Cabrero, reported a predominance of *T. canis* eggs in Talca, with a frequency of 14%, and eggs from the Ancylostomatidae family in Cabrero, with frequency of 43%. Notably, in both studies *T. vulpis* ranked second in frequency with percentages ranging from 5 to 12.9% (54, 55). There was no statistically significant difference between parasitosis and dogs going outside their house freely (*p* > 0.05). Over half of the surveyed pets regularly went outside, contrasting with 27% reported in urban area of the Coquimbo region, Chile, but similar to the 50% reported in rural areas in the same region (56). The absence of a statistically significant difference between parasitosis and factors such as deworming or outdoor activities habits may be attributing to the limitations of this research. A limitation of the study could be the selection bias that may have occurred during the selection of the participants by the pet owners. Furthermore, the small number of samples analyzed (n = 39) could reduce the strength of the statistical analysis (57).

#### Parasites in dog stools collected in the environment

The frequency of one or more parasitic elements in these samples was 48.4% in spring and 40% in autumn, with no statistically significant differences between the two periods (*p* > 0.05). These frequencies were higher than those reported in a similar study conducted in Santiago de Chile, where 31.7% of the samples contained parasitic elements (58).

In our study the parasitic species did not vary significantly between the two sampling periods, with *T. vulpis* and *U. stenocephala* being the most frequently observed species, ranging from 23.5 to 30% and from 14.1 to 16.8%, respectively. In a study carried out in urban areas of Niebla and Corral, it was also found that these two species were the most common, with a difference in the frequency of observation, since in this study the percentages for *T. vulpis* ranged from 50.7 to 54.8%, while for *U. stenocephala* were between 65.7 and 83.4% (15). Additionally, our study detected *Toxascaris leonina* during the spring at a frequency of 2.1%, consistent with previous studies in the Los Ríos region, which reported frequencies of 3.3–8.0% (59, 60). It has also been documented in other areas in the country, such as Los Angeles (Chile), where it was detected in 1.33% of the samples (13), and in Santiago (Chile), where it was observed in 7.7% of the samples (58, 61). The frequency of protozoan parasites with zoonotic potential where only the presence of *G. duodenalis* cysts was found in 1.1% in spring and 2.4% in autumn. In various studies carried out in Italy, Germany, Poland, Colombia and Mexico, it has been seen that the prevalence of these parasite in dog feces ranges from 0 to 25% (61–66). In general, the presence of protozoan parasites such as *G. duodenalis* in dog feces is underestimated (66–68). This could be attributed to several factors, as the intermittent elimination of infective stages of parasites can lead to false negatives when observing an isolated sample microscopically (61, 65).

#### OneHealth conclusions

In summary, our study identified a 28% prevalence of intestinal parasites and a 33% prevalence of anti-*T. canis* antibodies in humans; also, a 26% prevalence in owned dogs and 44% frequency of parasites in dog stools collected from the street during spring and fall. Additionally, up to 30.5% (spring and fall) of soil samples from parks in the study area was found to contain parasitic elements. Thus, using a OneHealth approach, our study highlights that the prevalence of parasites in humans is influenced non only by human-to-human transmission, but also and significantly, by zoonotic and environmental transmission. The most striking example of this is the high presence prevalence of anti-*T. canis* antibodies in humans (over 30%) alongside the detection of *Toxocara* sp. eggs in both parks soil and environmental dog feces. The absence of *T. canis* in the feces of owned dogs suggests environmental transmission as a primary pathway. In addition, zoonotic subtypes of *G. duodenalis* (A2 assembly) and *Blastocystis* sp. subtypes 1,2,3 and 4 were detected in humans, which have also been reported in dogs and cats, reinforcing the role of pets in parasite transmission.

This study highlights the importance of adopting a OneHealth approach to parasitology research. The complex ecology of parasitic organisms demands an integrated perspective to fully understand their transmission pathways and develop effective control strategies. By emphasizing the interconnectedness of human, animal, and environmental health, we aim to contribute to the management and mitigation of this persistent public health issue.

## Data Availability

The data presented in the study are deposited in NCBI repository, accession number PRJNA1231441, https://www.ncbi.nlm.nih.gov/bioproject/1231441.
