# Supplementary material for: High parasite prevalence driven by the human-animal-environment interface: a One Health study in an urban area in southern of Chile
Source: Front Vet Sci. 2025 Mar 21;12:1536861. doi: 10.3389/fvets.2025.1536861 (PMC11969455; doi:10.3389/fvets.2025.1536861)
Supplement: Supplementary file 1 [file Table_1.DOCX]

Table of contents

[SURVEY "Relationship between human health, the environment and animals. An integrated vision for a comprehensive understanding of health" 2](#_Toc182562767)

[Description of the NGS technique and primers 6](#_Toc182562768)

[Frequency (%) of observation of parasites taxa in dog feces collected from the environment, classified by sector (A, B or C) and by season (Spring= S; Autumn = Au) 7](#_Toc182562769)

# SURVEY "Relationship between human health, the environment and animals. An integrated vision for a comprehensive understanding of health"

Name___________________________________

Age__________ Sex________

Adress___________________________________

City_________________

Phone number____________________

Email________________________

1. What is their level of schooling?

- Basic education
- Secondary education
- Higher education (technical or university)
- None

1. What is your trade/job?

__________________________________________

1. What is the approximate family income?

- $0-300,000
- $300.001-600.000
- $600.001-1.000.000
- More than $1,000,000

1. Do you have one or more of the following chronic conditions? (You can select more than one alternative)

- Diabetes mellitus
- High blood pressure
- Asthma
- Rheumatoid arthritis
- Chronic kidney failure
- Other:________________________________

1. Why do you visit CECOSF?

- Adult Preventive Medicine Exam (EMPA)
- Well-Child Check-Up
- Cardiovascular control
- Pregnancy control
- Chronic disease management
- Other:________________________________

1. Do you do one or more of the following outdoor activities? If so, please indicate which ones (You can select more than one alternative):

- Cycling
- Hiking
- Fishing
- Running
- Other:_________________

1. How often do you do outdoor activities?

- Never
- Once a week
- 2-3 times a week
- 4-6 times a week
- Every day
- Other:________________________________

1. Have you had one or more of these signs/symptoms in the past three months? (You can select more than one alternative)

- Nausea and/or vomiting
- Diarrhea
- Dolor abdominal
- Constipation
- Bloating
- Gastric acidity
- Blood in the stool

**With respect to housing and cohabitants,**

1. Regarding your home:

- Number of rooms in use:
- Number of bedrooms:
- Number of people:
- Number of beds in use:

1. Do you live with minors at home?

- Yes
- No

10.1- If you answered yes to the previous question, please indicate below the age(s) of the minor(s):

_________________________________________

10.2- How many times a week do the minors you live with go to the park/square near your home?

- Never
- Once a week
- 2-3 times a week
- 4-6 times a week
- Every day
- Other:________________________

1. Do you have pets in your home?

- Yes
- No

11.1- If your answer to the previous question was yes, indicate what and how many pets you have:

- Dogs_____
- Cats______
- Other (indicate which) ____________________

11.2- Do your pets usually go outside and/or have contact with other pets with an owner and/or on the street?

- Yes
- No

11.3- Where do your pets defecate? (You can select more than one option.)

- Sandbox
- In the garden/courtyard
- On the street
- Other:________________________

11.4- Have you ever dewormed your pet(s)? If so, when was the last time I deworm it?

- Between 0-3 months ago
- Between 3-6 months ago
- Between 6 months to 1 year
- Over a 1 year ago
- Never

1. Do you own a garden or patio in your home?

- Yes
- No

1. What is your stool elimination system?

- Septic tank
- Sewer system
- Other:_________________________________

1. How do you remove trash from your home? (You can select more than one option.)

- Garbage collection truck
- Burning of waste
- Other:_________________________________

1. Have you or anyone you live with ever been under parasitological treatment?

- Yes, when?: __________________________
- No

1. Have you been abroad in the last three years?

- Yes
- No

16.1- If your answer was yes, indicate which country you went to:

_______________________________________

1. Do you live or have you lived in a plot or field (rural area) in the last 24 months?

- Yes
- No

17.1- If your answer was yes, indicate what your source of water was in that place:

- Safe rural drinking water
- Well water
- Rainwater
- Creek water
- Other:_________________________________

1. In the last 12 months have you only consumed drinking water?

- Yes
- No

18.1-If your answer was negative, indicate why you consumed water from another source:

________________________________________

_____________________________________

# Description of the NGS technique

A methodology combined with Trimomatic (v0.39; Bolger, Lohse, and Usadel 2014) and PRINSEQ (v0.20.4) (Schmieder and Edwards 2011) was used to process the sequences. First, Illumina adapter sequences were removed and readings were filtered by quality, retaining only those with an average Phred value ≥ Q30. Poor quality bases and ambiguous sequences (Ns) were also removed. For ASV (Amplicon Sequence Variants) inference, the DADA2 package in R (Callahan et al. 2016) was used, which includes error estimation by base, de-replication, ASV inference, paired read concatenation and chimera removal. Taxonomic mapping was performed with the DADA2 pipeline, using the RDP Naive Bayesian Classifier algorithm (Wang et al. 2007) and the SILVA v138 database (Quast et al. 2012)

The sequences of the ASVs that were not taxonomically assigned were aligned with the ASVs assigned to *Blastocystis* sp. and a phylogenetic tree was constructed using a sequence of *Giardia* sp. as an outgroup. Subsequently, the NA sequences identified by phylogeny as *Blastocystis* were corroborated by BLAST against the NCBI database. Finally, to determine the subtype(s) of *Blastocystis* present(s) in the samples, representative sequences of each of the subtypes available in the NCBI were selected, and after alignment, a Bayesian tree was generated, which included the ASVs assigned to *Blastocystis*, the representative subtypes of the NCBI and the sequence of *Giardia* sp. as an outgroup. ASVs were assigned to a subtype according to their clade, and the proportions of ASVs in each sample were represented by abundance barplots using R's ggplot package.

| **Parásito** | **Gen** | **Código GenBank** | **Secuencia de oligonucleótidos**  **5' a 3'** |
| --- | --- | --- | --- |
| *Blastocystis* spp. | 18s | XM_013038637.1 | FS-GGAGGTAGTGACAATAAATC  AS-GCTTTCGCACTTGTTCATC |
| *Giardia duodenalis* | β-giardin | XM_001705373.1 | FS-GAACGAGATCGAGGTCCG  AS-CTCGACGAGCTTCGTGTT |

| **Sector** | *Trichuris vulpis* | | *Uncinaria stenocephala* | | *Toxocara* sp. | | *Toxascaris leonina* | | Capillaridae gen. sp. | | *Giardia lamblia* | |
| --- | --- | --- | --- | --- | --- | --- | --- | --- | --- | --- | --- | --- |
|  | S | Au | S | Au | S | Au | S | Au | S | Au | S | Au |
| A  (Spring n=49; Autumn n=24) | 30.6  (n=15) | 41.7  (n=10) | 18.4  (n=9) | 33.3  (n=8) | 4.1  (n=2) | 16.7  (n=4) | 4.1  (n=2) | - | 6.1  (n=3) | - | - | 4.2  (n=1) |
| B  (Spring n=25;  Autumn n=24) | 32.0  (n=8) | 16.7  (n=4) | 12  (n=3) | - | - | - | - | - | - | - | 4  (n=1) | - |
| C  (Spring n=21;  Autumn n=37) | 28.6  (n=6) | 16.2  (n=6) | 19.1  (n=4) | 10.8  (n=4) | 9.5  (n=2) | - | - | - | - | - | - | 2.7  (n=1) |
| Total | 30.53%  (n=29) | 23.53%  (n=20) | 16.84%  (n=16) | 14.12%  (n=12) | 4.21%  (n=4) | 4.71%  (n=4) | 2.11%  (n=2) | - | 3.16%  (n=3) | - | 1.05%  (n=1) | 2.35%  (n=2) |

# Frequency (%) of observation of parasites taxa in dog feces collected from the environment, classified by sector (A, B or C) and by season (Spring= S; Autumn = Au)
